# Respiratory carriage of hypervirulent *Klebsiella pneumoniae* by indigenous populations of Malaysia

**DOI:** 10.1186/s12864-024-10276-4

**Published:** 2024-04-17

**Authors:** Souradeep Das, Anish K. Pandey, Denise E Morris, Rebecca Anderson, Victor Lim, Chong Chun Wie, Ivan Kok Seng Yap, Ahmed Ghazi Alattraqchi, Hafis Simin, Ramle Abdullah, Chew Chieng Yeo, Stuart C. Clarke, David W. Cleary

**Affiliations:** 1 Faculty of Medicine, Institute for Life Sciences, University of Southampton, Southampton, UK; 2 NIHR Southampton Biomedical Research Centre, University Hospital Southampton Foundation NHS Trust, Southampton, UK; 3 School of Medicine, International Medical University, Kuala Lumpur, Malaysia; 4 Institute for Research, Development and Innovation, International Medical University, Kuala Lumpur, Malaysia; 5 School of Pharmacy, Monash University Malaysia, Bandar Sunway, Selangor Malaysia; 6 Centre for Research in Infectious Diseases and Biotechnology (CeRIDB), Faculty of Medicine, Universiti Sultan Zainal Abidin, Medical Campus, 20400 Kuala Terengganu, Terengganu Malaysia; 7 Faculty of Applied Social Sciences, Universiti Sultan Zainal Abidin, Gong Badak Campus, 21300 Kuala Nerus, Terengganu Malaysia; 8 Centre of Excellence in National Indigenous Pedagogy, Institute of Teacher Education Tengku, Ampuan Afzan Campus, Pahang, Malaysia; 9 Global Health Research Institute, University of Southampton, Southampton, UK; 10 Institute of Microbiology and Infection, College of Medical and Dental Sciences, University of Birmingham, University of Birmingham, UK

**Keywords:** *Klebsiella pneumoniae*, Malaysia, Antimicrobial resistance, ESBL, Hypervirulent

## Abstract

*Klebsiella pneumoniae* is a Gram-negative Enterobacteriaceae that is classified by the World Health Organisation (WHO) as a Priority One ESKAPE pathogen. South and Southeast Asian countries are regions where both healthcare associated infections (HAI) and community acquired infections (CAI) due to extended-spectrum β-lactamase (ESBL)-producing and carbapenem-resistant *K. pneumoniae* (CRKp) are of concern. As *K. pneumoniae* can also exist as a harmless commensal, the spread of resistance genotypes requires epidemiological vigilance. However there has been no significant study of carriage isolates from healthy individuals, particularly in Southeast Asia, and specially Malaysia. Here we describe the genomic analysis of respiratory isolates of *K. pneumoniae* obtained from Orang Ulu and Orang Asli communities in Malaysian Borneo and Peninsular Malaysia respectively. The majority of isolates were *K. pneumoniae* species complex (KpSC) 1 *K. pneumoniae* (*n* = 53, 89.8%). Four *Klebsiella variicola* subsp. *variicola* (KpSC3) and two *Klebsiella quasipneumoniae* subsp. *similipneumoniae* (KpSC4) were also found. It was discovered that 30.2% (*n* = 16) of the KpSC1 isolates were ST23, 11.3% (*n* = 6) were of ST65, 7.5% (*n* = 4) were ST13, and 13.2% (*n* = 7) were ST86. Only eight of the KpSC1 isolates encoded ESBL, but importantly not carbapenemase. Thirteen of the KpSC1 isolates carried yersiniabactin, colibactin and aerobactin, all of which harboured the *rmpADC* locus and are therefore characterised as hypervirulent. Co-carriage of multiple strains was minimal. In conclusion, most isolates were KpSC1, ST23, one of the most common sequence types and previously found in cases of *K. pneumoniae* infection. A proportion were hypervirulent (hv*Kp*) however antibiotic resistance was low.

## Introduction

For indigenous communities across the globe there is no doubt that there continues to exist disparities in susceptibility to, and burden from various diseases [1]. Marginalisation, resulting in inadequate access to health and social care infrastructure, often results in these communities being at risk of higher infant mortality and reduced life expectancy [1]. Whilst the term ‘indigenous’ is not proscribed as a term to encompass all such peoples, examples of ongoing burden in Canadian First Nations, Métis, and Inuit communities [2] and Australian Aboriginals [3] are all too easy to find. The Orang Ulu and Orang Asli are considered the indigenous peoples of Malaysia, living traditional lifestyles with similar socio-economic and health burdens [4, 5]. Typically, these communities live in poverty which translates into a life expectancy difference of ∼ 20 years compared to the rest of the Malaysian population [6]. Given the distinct challenges that indigenous communities encounter, it is therefore critical that specific and targeted attention is given to them with respect to the infectious disease burden they may face [7]. To that end we draw our attention to *Klebsiella pneumoniae*.

*K. pneumoniae* is a Gram-negative Enterobacteriaceae that is classified by the World Health Organisation (WHO) as a Priority One ESKAPE pathogen [8]. As a major threat to public health, it remains a common nosocomial pathogen causing serious healthcare associated infections (HAI) including bacteraemia, pneumonia, and sepsis. Similarly to many infectious diseases the burden is shouldered most by those at the extremes of age, as well as the immunocompromised [9].

The *K. pneumoniae* species complex (KpSC) comprises seven phylogroups, based on genomic relatedness: *K. pneumoniae* (KpSC1), *K. quasipneumoniae* subsp. *quasipneumoniae* (KpSC2), *K. variicola* subsp. *variicola* (KpSC3), *K. quasipneumoniae* subsp. *similipneumoniae* (KpSC4), *K. variicola* subsp. *tropica* (KpSC5), *K. quasivariicola* (KpSC6), and *K. africana* (KpSC7) [10, 11]. Whilst KpSC1 is the predominant phylogroup responsible for global disease [12], this diversity in the species complex has hampered efforts to generate effective vaccines and therapeutics. Moreover, the emergence of multidrug resistance to carbapenems, broad-spectrum β-lactams, fluoroquinolones, and aminoglycosides pose a significant challenge. Consequently, genomic surveillance, to monitor for example the spread of problematic clonal complexes such as CC258 [13], is an absolute requirement for this important human pathogen.

As *K. pneumoniae* can also exist as a harmless commensal, the prevalence of resistant genotypes within these reservoirs also requires epidemiological vigilance. To date there has been no significant study of respiratory carriage isolates from healthy individuals in Southeast Asia, and especially Malaysia. However, broadly, South and Southeast Asian countries are regions where both HAI and community acquired infections (CAI) due to extended-spectrum β-lactam (ESBL)-producing and carbapenem-resistant *K. pneumoniae* (CRKp) are of particular concern [14–16]. A recent study of blood stream infections, involving seven major hospitals in South and Southeast Asian countries, found 17% of *K. pneumoniae* strains were carbapenemase producers and 47% of the strains carried markers for ESBL. Additional concerns were raised due to the high prevalence of aerobactin synthesis locus (*iuc*) in association with ESBL and/or carbapenemases [14].

Here we report the genomic epidemiology of *K. pneumoniae* that were taken during an all-age, upper respiratory tract carriage study in partnership with Orang Ulu communities in Sarawak, Malaysian Borneo and Orang Asli communities in Peninsular Malaysia.

## Methods

### Study population

Isolation of *K. pneumoniae* was done during an all-age, Orang Ulu community carriage study in April 2016 in Sarawak, Malaysian Borneo (isolates collected between 09/04/2016 and 01/05/2016) and a similar study in Orang Asli communities in Peninsular Malaysia in August 2017 (isolates collected between 01/08/2017 and 05/08/2017). These studies have both been published separately without the data presented herein [17, 18]. Briefly, in Sarawak, the largest state in Malaysian Borneo, four rural longhouse communities and one village were visited. These varied in both isolation and affluence, from Rumah Bana the most affluent and only 30 km from a nearby town, to Ba Marong which was located in dense forest 157 km from Long Lama. The reported ethnicities of the total population recruited for the initial study (*n* = 140) encompassed Iban, Kelabit, Kenyah and Penan. In Terengganu, situated in the north-east of peninsular Malaysia two villages were visited from which *n* = 130 participants were recruited. All participants were reported as Orang asli. There were no exclusion criteria for recruitment. Participants were asked to complete a questionnaire requesting demographic data and medical history such as recent respiratory tract infections, and any history of use of antimicrobials and immunisation status.

### *Klebsiella sp.* isolates

Participants received whole mouth (WMS), oropharyngeal (OP), nasopharyngeal and/or nasal swabbing (N). Swabs were either rayon tipped Transwab® Pernasal Amies with charcoal (Medical Wire and Equipment, Corsham, UK) for paediatric NP, or viscose tipped sterile Amies swabs with charcoal (Deltalab, Chalgrove, UK) for WMS, OP and N. Initial culture, performed at the University of Southampton (UK) following transportation, was done as outlined previously [17, 18], but briefly swabs were plated onto multiple media for the purposes of isolating common respiratory pathobionts. These included: CBA (Columbia blood agar with horse blood), CHOC (Columbia blood agar with chocolated horse blood), CNA (Columbia Blood Agar with Colisitin and Naladixic Acid), BACH (Columbia Agar with Chocolated Horse Blood and Bacitracin), GC (Lysed GC Selective Agar) and *Pseudomonas* CFC Selective agar (all Oxoid, UK). Suspected *K. pneumoniae*, those being mucoid coliforms on CBA plates, were sub-cultured onto CLED agar (Oxoid, UK) for confirmation using matrix-assisted laser desorption ionization time-of-flight mass spectrometry (MALDI-TOF) using a Microflex® LRF (Bruker, UK). Where multiple isolates were taken from one individual these were included regardless of the niche from which they were taken i.e., if a *Klebsiella* sp. was isolated from the nose and mouth of a participant, both were stored. These were used to determine frequency of multi-strain carriage based on genomic analyses.

### Antibiotic susceptibility

*K. pneumoniae* was spread over Mueller-Hinton agar plates (MH, Oxoid, UK). Antibiotic discs (Oxoid, UK) were added before pates were incubated at 35 ± 1^°^C for 18 h (± 2 h). *K. pneumoniae* were tested with cefotaxime (5 µg), ciprofloxacin (5 µg), meropenem (10 µg) and ceftazidime (10 µg) antibiotic discs. Susceptibility was determined against EUCAST Clinical Breakpoint guidelines v6.0.

### DNA extraction

DNA was extracted from a sweep of growth using a QIAmp DNA Mini kit (Qiagen, UK), as per the manufacturer’s instructions. The concentration of genomic DNA was determined using Qubit 2.0 fluorometric quantification (Thermo-Fisher, UK).

Whole Genome Sequencing: Library preparation was done using the Nextera XT DNA kit (Illumina, UK) following the manufacturer’s instructions. Briefly DNA was quantified with Qubit fluorometric quantification (ThermoFisher, UK) and diluted to 0.2 ng/µl. Following dilution, 1 ng DNA was tagmented with the Nextera XT transposome. Tagmented libraries were amplified with 12 cycles of PCR and dual-indexed primers. Libraries were cleaned and size selected using 0.5× volume AMPure XP beads (Beckman Coulter™, Fisher Scientific). Library concentrations were normalised using the bead-based normalisation protocol implemented in the Nextera XT kit, then normalised libraries were pooled in equal volumes. Sequencing to generate 2 × 250 bp paired end reads was done on a MiSeq (Illumina, UK) using the 500 cycle v2 reagent kit.

### Sequence quality control and assembly

FASTQC v0.11.3 (https://github.com/s-andrews/FastQC) with MULTIQC v1.9 [19] was used to perform quality assessment on paired-end reads. Kraken v7.3.0 [20], a kmer-based taxonomy classification tool, was used to check for contamination and confirm the isolates were *K. pneumoniae*. Paired-end reads were trimmed using trimmomatic v0.39 [21] and *de novo* assembled using SPAdes v3.14.1 [22]. Assemblies were iteratively improved using pilon v1.23 [23] and then Quast v5.0.2 was used to evaluate the assemblies [24].

### Phylogenomics

Snippy v4.4.1 (https://github.com/tseemann/snippy), using snippy-multi, was used to generate a core SNP-based alignment using *Klebsiella pneumoniae* subsp. *pneumoniae* Ecl8 (accession: GCA_000315385.1) as a reference. The subsequent alignment was character-corrected using snippy-clean before recombination regions were identified and removed using the accompanying run_gubbins.py and snp-sites scripts, before FastTree was used to generate a phylogenetic tree using the GTR model of nucleotide substitution.

### MLST, virulence and antibiotic gene identification/detection

Kleborate v1.0.0 [25, 26] was used to define *K. pneumoniae* species complex (KpSC), MLST, serotype predictions of K (capsule) and O antigen as well as a range of ICE*Kp* and plasmid associated virulence loci in addition to antimicrobial resistance determinants.

### Data analysis

All analysis was done in R version 3.6.0 (2019-04-26) using RStudio version 1.2.1335 [27, 28] with graphics built using the grammar of graphics package, ggplot [28]. The phylogenetic tree was visualised using the r package treedataverse, specifically ggtree [29].

## Results

### *K. pneumoniae* colonised participants

A total of sixty-seven isolates (from forty-eight individuals) were isolated from the two previously described carriage studies. Participant demographics and location of those from whom *K. pneumoniae* were isolated are shown in Table 1. Children represented only 8.4% (*n* = 4/48) of this cohort, with most isolates recovered from those between the ages of 18 and 64 (35.4%; *n* = 17). There were more female participants (54.2%) than male. Forty-two participants came from the locations in Sarawak, with the majority (37.5%; *n* = 18) taken during the visit to Rumah Numpang, an isolated longhouse community located in Sebauh, Bintulu.

**Table 1:** Demographics of participants from whom *K. pneumoniae* were isolated

	Group	N (%)
**Age (Years)**	< 5	1 (2.1)
	5–17	3 (6.3)
	18–49	17 (35.4)
	50–64	16 (33.3)
	65+	10 (20.8)
	NA	1 (2.1)
**Gender**	M	16 (33.3)
	F	26 (54.2)
	NA	2 (4.2)
**Location**		
Peninsular Malaysia	Kampung Sungai Pergam	4 (8.4)
	Kampung Berua	2 (4.2)
Sarawak	Rumah Numpang	18 (37.5)
	Rumah Bana	12 (25.0)
	Long Nen	5 (10.4)
	Long Kerangan	3 (6.3)
	Ba Marong	2 (4.2)
	Kampung Sebir	2 (4.2)

### Genomics of *K. pneumoniae* isolates

Eight isolates were excluded from genome analysis after failing assembly quality checks. Four had N50s < 1Mbp, three had N50s < 1Mbp and genome sizes > 6Mbp and a final isolate had a genome size > 6Mbp and the assembly was highly fragmented (> 1000 contigs). Of the remaining fifty-nine isolates the majority (89.8%; *n* = 53) were KpSC1 *K. pneumoniae*, with four *Klebsiella variicola* subsp. *variicola* (KpSC3) and two *Klebsiella quasipneumoniae* subsp. *similipneumoniae* (KpSC4).

**Fig. 1:**
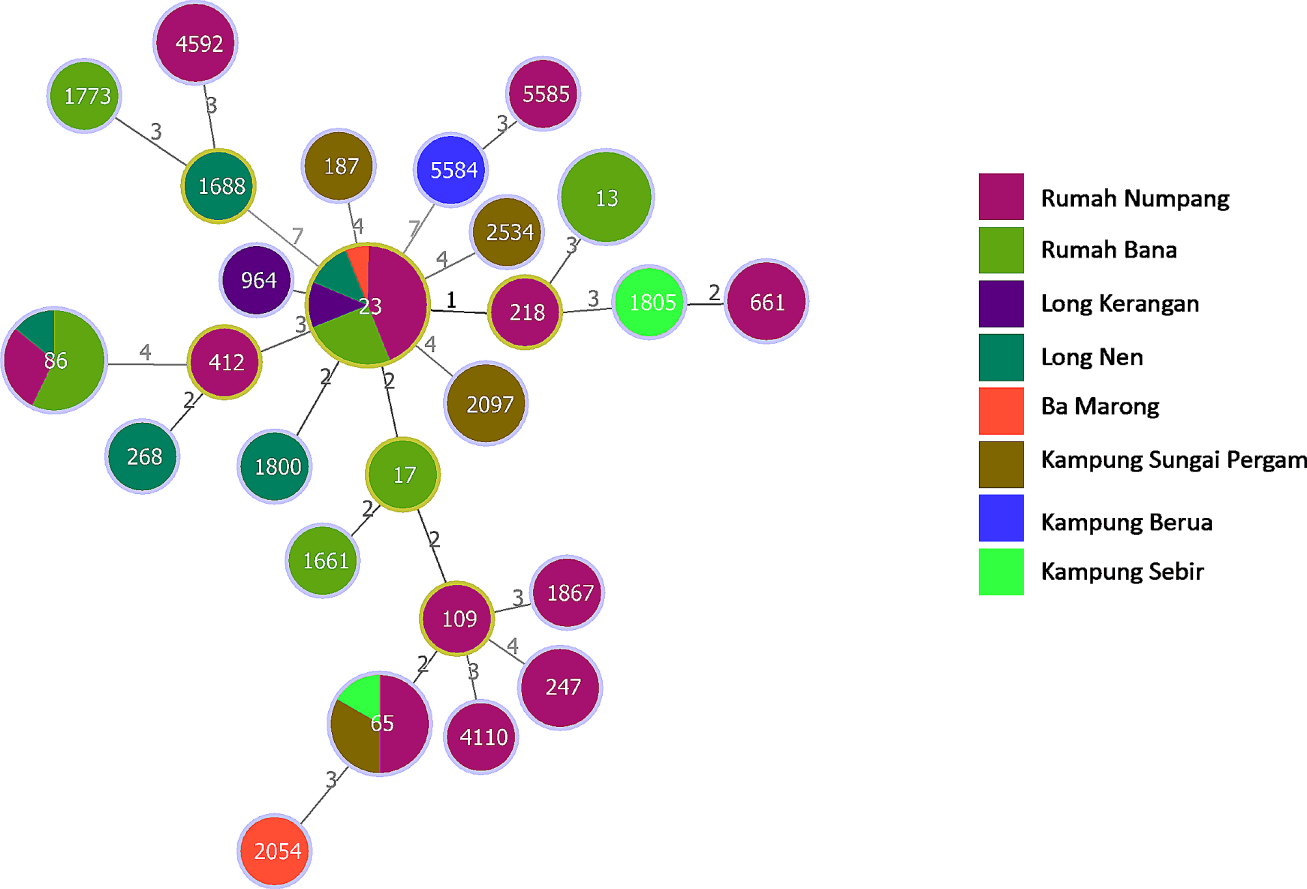
Minimum-spanning tree generated using goeBurst and MLST. Node numbers are STs and edge numbers represent allelic differences. Node size is proportional to number of isolates belonging to that ST and are coloured by location of isolation

Twenty-six unique MLSTs were identified (Fig. 1). The majority of the STs were only observed once, with most isolates (*n* = 29/59, 49.2%) belonging to only three STs: ST23 (*n* = 16, 27.1%), ST86 (*n* = 7, 11.9%) and ST65 (*n* = 6, 10.2%). ST5584 and ST5585 were novel to this study.

Fourteen capsule (K) types were identified. Of these KL1 accounted for most at 27.1% (*n* = 16/59), followed by KL2 (18.6%, *n* = 11/59) and KL3 (8.5%, *n* = 5/59). For twelve (20.3%) isolates no accurate capsule locus could be identified. It is likely these ‘unknown’ loci were due to lack of assembly contiguity or low/absence of coverage given that on average the locus evidence was present on between six and seven contigs and was typically missing between five to six genes.

### Virulence and antibiotic resistance

**Fig. 2:**
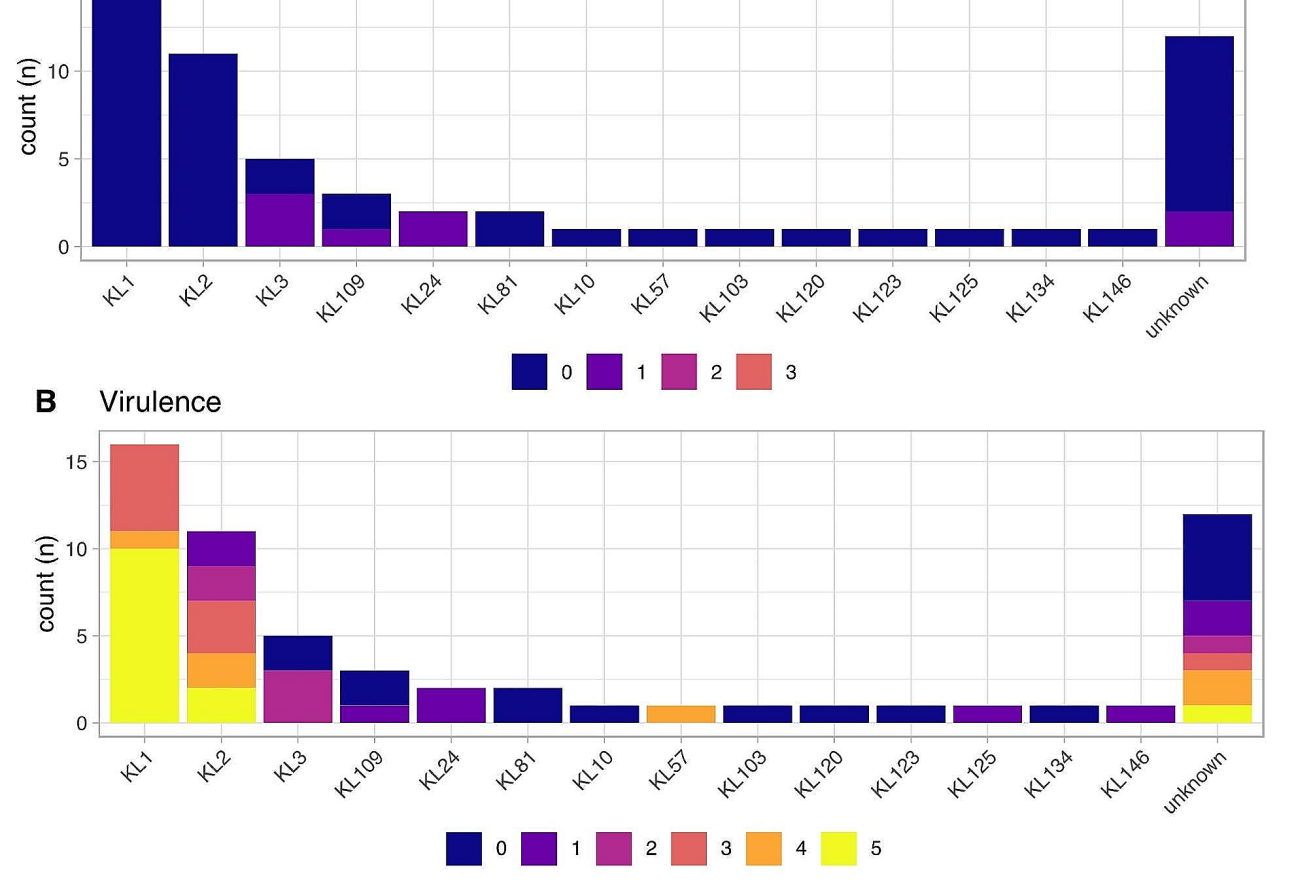
Antibiotic resistance (**A**) and acquired virulence (**B**) scores for isolates with known capsular types. Resistance scores are on a scale of 0 (low) to 3 (high), and virulence 0 (low) to 5 (high). Resistance scores of 0 indicates no ESBL and no carbapenemase, 1 indicates the presence of an ESBL but still without carbapenemase, 2 indicates the presence of a carbapenemase without colistin resistance and 3 a carbapenemase with colistin resistance. Scores of 0/1 are irrespective of colistin resistance with scores of 2/3 irrespective of the presence of an ESBL. Virulence scores are based on the presence of yersinibactin (*ybt*), colibactin (*clb*) and/or aerobactin (*iuc*). A score of 0 indicates none of these genes were found with a score of 5 showing all three were present. Scores of 1–4 indicate the following: 1 - yersiniabactin only, 2 - yersiniabactin and colibactin (or colibactin only), 3 aerobactin only, 4– aerobactin and yersiniabactin. Bars are ordered by frequency of capsule type

Acquired virulence gene and resistance scores are shown in Fig. 2. Only eight isolates, three of which were ST13 KL3, had a resistance score (Fig. 2A), and these were all ‘1’ indicating they were ESBL producers but without carbapenemases. This is in keeping with the phenotypic antibiotic resistance testing where all isolates were susceptible to all antibiotics tested. Resistance scores (Fig. 2B) were more varied. Those with the highest scores (5; indicating the presence of yersiniabactin, colibactin and aerobactin) were KL1 ST23 (*n* = 10: ybt 1 *ICE*Kp10, clb 2, iuc 1) and KL2 ST65 (*n* = 2: ybt 17 *ICE*Kp10, clb 3, iuc 1).

**Fig. 3:**
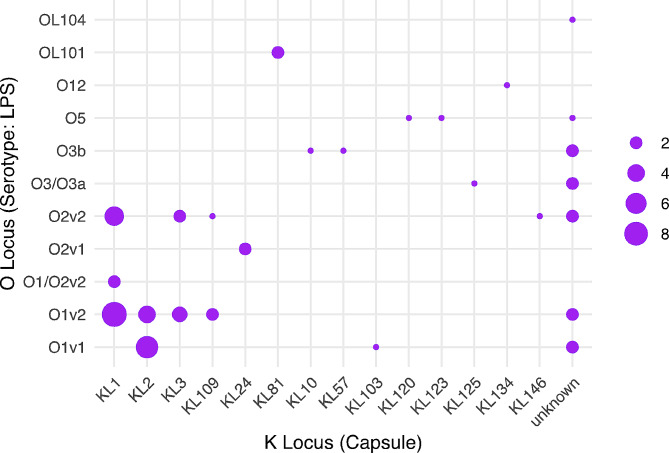
Correlation between capsule (K) and serotype (O) loci. Each point displays the number of isolates within each K/O group. Most isolates were characterized by O1 or O2 polysaccharide which were found in KL1, KL2, KL3, KL109 and KL124 (bottom left quadrant)

The distribution of O serotypes is shown in Fig. 3. O1/02 serotypes accounted for 66% of isolates (*n* = 39) and were found associated with six capsule types (K1, K2, K3, K24, and K109).

### Phylogeny of *K. pneumoniae* isolates

**Fig. 4:**
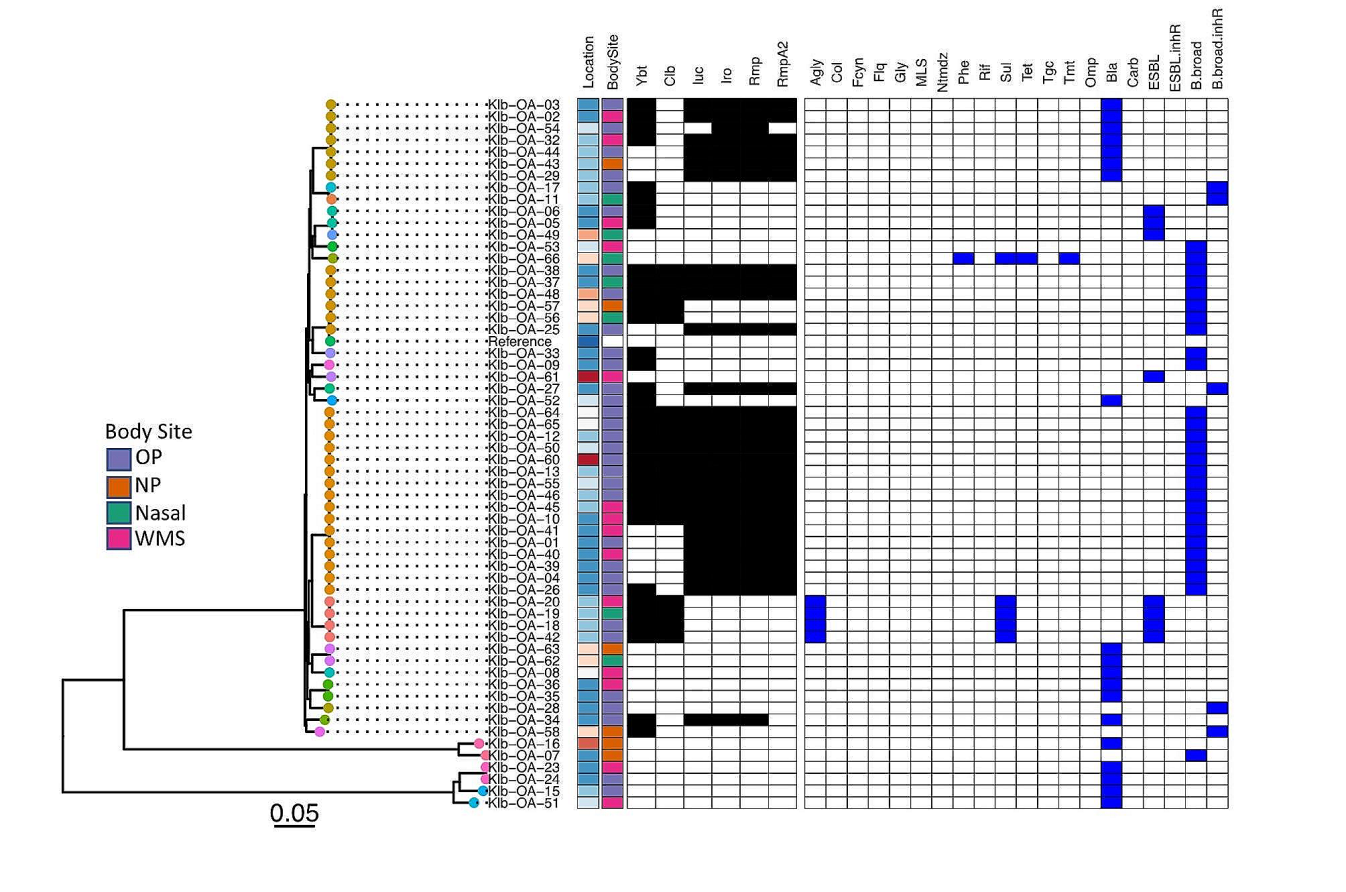
Phylogeny of *K. pneumoniae.* Phylogenetic tree based on core-genome SNPs and constructed using FastTree (GTR + GAMMA). Leaves are colored by ST. The presence of virulence genes are shown in black, with antibiotic resistance markers in blue (white indicates absence). Virulence: Ybt yersiniabactin, Clb colibactin, Iuc aerobactin, Iro salmochelin, Rmp and RmpA2 hypermucoidy. Antibiotics: Agly aminoglycosides, Col colistin, Fcyn Fosfomycin, Flq fluoroquinolones, Gly glycopeptides, MLS macrolides, Ntmdz nitroimidazoles, Phe phenicols, Rif rifampin, Sul sulfonamides, Tet tetracyclines, Tgc tigecycline, Tmt trimethoprim, Omp osmoporin mutations, Bla beta-lactamases, Carb carbapenemase, ESBL extended spectrum beta-lactamases, ESBL(inhR) extended spectrum beta-lactamases with resistance to beta-lactamase inhibitors, B.broad beta-lactamases, B.broad.inhR beta-lactamases with resistance to beta-lactamase inhibitors

The three KpSCs, KpSC1, 3 and 4, are clearly visible on the isolate phylogeny with KpSC1 being the largest cluster (Fig. 4). No distinction between geographic location or body site sampling can be seen. All isolates which had a virulence score of 5 (indicated by black squares for yersiniabactin, colibactin and aerobactin) also harboured the *rmpADC* locus (*n* = 13) conferring a hypermucoid phenotype and would therefore be classified as hypervirulent *K. pneumoniae* (hv*Kp*). These isolates in particular only harboured the chromosomally encoded β-lactamase (B.broad column, Fig. 4) and are therefore not considered a problem with respect to antimicrobial resistance. Moreover, the presence of resistance genes overall was very limited. Only eight isolates had ESBL of which only two (both KL24 ST661) had any of the virulence genes, which in this case was *ybt*. We also note the high proportion of isolates generally encoding *rmpA/A2* (*n* = 29/60). All were KpSC1, with KL1 (*n* = 16), KL2 (*n* = 11) and KL57 (*n* = 2) being the only capsule locus types associated. All KL1 were ST23, whereas KL2 was split reasonably evenly between ST86 (*n* = 7) and ST65 (*n* = 4). All isolates were from adults, with the majority (*n* = 22/29, 76%) coming from those over the age of 50-years-old. There was no gender separation with those coming from females and males in the same proportion (1:1.6) mirroring study recruitment. All but two isolates were from oropharyngeal/whole mouth swabs.

### Carriage of multiple *K. pneumoniae* strains

We were particularly interested in the question of multiple strain carriage. Of the 18 participants from whom multiple isolations of *K. pneumoniae* were made, nine had isolates from both WMS and OP swabs, three from both OP and N and two from a N and NP sample. This excludes four participants for whom the genome assemblies of one or both isolates were not analysed as outlined above. Of those remaining, only two participants exhibited a multiple-strain carriage phenotype: an 80-year-old female from Rumah Bana had an ST86 KL2 in her OP sample and an ST17 with unknown capsule type in her nasal sample, and a 48-year-old female from Long Nen harboured a ST1800 KL109 and a ST268 unknown capsule type in OP and WMS samples respectively.

## Discussion

Countering the spectre of rising antimicrobial resistance in *K. pneumoniae* requires continued genomic epidemiological vigilance. Whilst there are efforts to fill the gap in knowledge regarding strains of this important human pathogen from Malaysia, specifically carbapenemase producers isolated in clinical settings [30, 31], there is a paucity of information related to carriage. Therefore, to add to this burgeoning field, we present the first genomic study of *K. pneumoniae* isolates from respiratory tract carriage, with a focus on an understudied and marginalised community of Malaysia. We highlight the high frequency of hypervirulent strains with, at the present time, minimal repertoires for antimicrobial resistance.

With limited regional data it is difficult to compare our isolates either with those from elsewhere in Malaysia, or within the Southeast Asian region, particularly with respect to respiratory carriage. Nevertheless, there are useful comparisons which may be made. For example, our finding that 19% of our isolates were hv*Kp* is reasonably similar to the ∼ 10% identified in studies of gut colonisation in a Taiwanese hospital setting and a study of healthy Chinese adults [32, 33]. In the latter study, KL1 and KL2 were also the most frequent capsule types observed, and that included in those Chinese adults residing in Malaysia [34]. This dominance of KL1/2 has also been noted in Singapore [35]. In keeping with the Singaporean epidemiology, the most common sequence type from our study was also ST23, which has been found to be associated with hypervirulence and antimicrobial resistance of *K. pneumoniae* in South East Asia [36] and previously identified as a common cause of disease in a collection of isolates from a Malaysian teaching hospital [37]. There are however important contrasts to note from the latter clinical study. There, *hv*Kp accounted for a significantly greater proportion of isolates (38%), and MDR/ESBL was also more prevalent (31.9 and 27.8% respectively) [37]. Indeed, whilst ST23 was shared, ST22 and ST412, the next two most common STs were not found in the present study [37]. The increased proportion of ESBL-producers in clinical samples was also observed in a two year study of hospital blood stream infections [38]. Here, 53% of isolates were classed as ESBL-producers (*n* = 45/303) [38]. Our study provides a further stark contrast to the 87% prevalence of carbapenem-resistant *K. pneumoniae* clinical isolates that were observed over the course of one year at the University Malaya Medical Centre, Kuala Lumpur [31]. Our study is however in keeping with the global picture of associations between hv*Kp* and antimicrobial resistance, in that those isolates which would be classed as hv*Kp* only had limited resistance, genotypically, to β-lactamas without ESBL and carbapenemases [25]. This reflects perhaps an important difference between carriage and clinical strains, as according to the most recent National Antibiotic Resistance Surveillance Report from 2020, antibiotic resistance has been rising in clinical strains in Malaysia [39]. Regardless of these distinctions, a recent surveillance study of MDR and hv*Kp* within low- and lower-middle income countries raised the importance of strain convergence [40]. This phenomenon whereby a hypervirulent and carbapenem-resistant strain arises was noted in 2015 in China [41]. As such, and for other examples since, Asia was flagged as a region where convergence was of particular concern [40]. Whilst Malaysia was not one of the three countries highlighted, the circulation of both hv*Kp*, as shown here, with the many examples above of clinical carbapenemase/ESBL-producing strains is cause for concern.

There are several limitations to this study. Firstly, the use of Illumina short-read sequencing and the resulting inability to accurately reconstruct K loci for all isolates would have been negated using long-read approaches. Further, no phenotypic analysis of capsule production was undertaken which would have clarified these discrepancies. The lack of accurate medical records makes extrapolation between the low AMR observed in these isolates and antibiotic use in these populations difficult. Our primary focus was respiratory carriage and therefore we do not know the carriage prevalence in the gut. This would be an important additional epidemiological reference point for future studies. Finally, our demographic is skewed towards an older population in only two locations. Despite these issues, perhaps the most important strength of our study is the focus on a marginalised, indigenous community. It is well documented that similar communities across the world are at increased risk of respiratory disease. Such examples include from the epidemiology of severe community acquired pneumonia in Australian Aboriginals [42] to increased incidence of invasive Group A Streptococci in First Nations populations in Alberta Canada [43] and *Staphylococcus aureus* disease in Native American individuals, where burden is many times the national average [44]. Whilst we have not determined *K. pneumoniae* disease burden in our Orang Ulu and Asli populations, this study is an important step in future attempts to do so.

In conclusion, we present the first study of carried respiratory *K. pneumoniae* from Malaysia. Currently, whilst there is clearly a reservoir of strains capable of causing disease, they at present do not harbour the genetics for resistance to therapeutic interventions.

## Data Availability

The datasets generated and/or analysed during the current study are available in the European Nucleotide Archive (ENA) under project PRJEB51966 with accession numbers ERX9079753 to ERX9079811.
